# DiGeorge Syndrome With Absence of Speech: A Rare Case

**DOI:** 10.7759/cureus.37745

**Published:** 2023-04-18

**Authors:** Srilakshmi K Jayaprakasan, Maymona E Nageye, Alveena Siddiqui, Gabriela Suero, Jorge Pimentel Campillo, Shaniah S Holder

**Affiliations:** 1 Pediatrics, Dr. B.R. Ambedkar Medical College and Hospital, Bengaluru, IND; 2 Internal Medicine - Pediatrics, Avalon University School of Medicine, Willemstad, CUW; 3 Pediatrics, Jinnah Sindh Medical University, Karachi, PAK; 4 Internal Medicine, CEDIMAT (Centros de Diagnóstico y Medicina Avanzada y de Conferencias Médicas y Telemedicina), Santo Domingo, DOM; 5 Medicine, American University of Barbados School of Medicine, Bridgetown, BRB

**Keywords:** absence of speech, management of a child with digeorge syndrome, digeorge syndrome with absence of speech, rare genetic disorder, digeorge syndrome

## Abstract

DiGeorge syndrome (DGS) is a rare genetic disorder caused by a deletion or abnormality of a small piece of chromosome 22. This condition can affect multiple organs in the body, including the heart, thymus, and parathyroid glands. While speech and language difficulties are common in individuals with DGS, the complete absence of speech is a rare presentation. This case report presents the clinical features and management of a child with DGS who presented with an absence of speech. The child underwent a multidisciplinary intervention approach, including speech and language therapy, occupational therapy, and special education, to improve their communication skills, motor coordination, sensory integration, academic performance, and social skills. The interventions resulted in some improvement in their overall function; however, speech improvement was not significant. This case report contributes to the literature on DGS by highlighting the potential underlying causes of speech and language difficulties in patients with this condition, and the possible etiologies that may lead to a complete absence of speech, which is a severe manifestation. It also emphasizes the importance of early recognition and intervention with a multidisciplinary approach to management, as early intervention can lead to better outcomes for patients with DGS.

## Introduction

DiGeorge syndrome (DGS), also known as velocardiofacial syndrome, is a rare genetic disorder inherited in an autosomal dominant fashion [1]. Dr. Angelo DiGeorge first described it in 1965 when a group of infants with a congenital absence of the thymus and parathyroid glands was found [1]. This syndrome is caused by a deletion in chromosome 22q11.2 and is characterized by abnormal development of the third and fourth pharyngeal pouches [2]. This syndrome classically presents with congenital thymic and parathyroid hypoplasia, which presents with immunodeficiency to viral and fungal pathogens and hypocalcemia due to hypoparathyroidism [2]. Other common manifestations include conotruncal heart malformations such as tetralogy of Fallot, dysmorphic facial features, including cleft palate and lip, low-set ears, and short palpebral features, and neurodevelopmental problems like autism spectrum disorder and attention deficit hyperactivity disorder (ADHD) [2].

The incidence of DGS is approximately one in 3,000-6,000 and continues to increase annually [3]. There are no significant risk factors in relation to gender or race since males and females of any ethnicity are equally affected but the risk of developing DGS is increased in relationships where at least one parent is affected [1]. The severity of symptoms ranges, and patients may not present typically, causing this disease to be overlooked and underdiagnosed without proper screening methods [3]. Developmental delay of the gross and fine motor skills as well as speech is a common manifestation of DGS and affects approximately 90% of patients [4].

Speech delay is considered in children whose development is below the norm for children of the same age [5]. In a study by Baylis et al., the authors found that 58.8% and 82.4% of the 17 adolescent participants with the 22q deletion met the criteria for speech delay and motor speech disorder, respectively [4]. Delays in speech may improve with speech therapy; however, in rare cases, patients have a complete absence of speech where there is no articulation of verbal expression. Absent speech occurs when there is no speech or language development, rendering patients nonverbal [6]. Complete absence of speech is a severe manifestation of DGS and has not been reported in the literature, with most cases having a delayed speech onset that improved with speech therapy. Herein, we present a case of a patient with DGS who had absent speech instead of the typical speech delay with no significant improvement despite speech therapy.

## Case presentation

A seven-year-old male child was brought to the psychiatry outpatient department of a tertiary care hospital with a complaint of an absence of speech. The patient was diagnosed with DGS at birth. On examination, the patient had dysmorphic facial features, including a small chin, a short nose, and widely spaced eyes. He had no spontaneous speech, and his vocalizations were limited to grunts, moans, and occasional cries. He had no signs of oral motor dysfunction and could produce a range of non-speech sounds, including clicks and whistles. He showed no signs of hearing loss on the audiological evaluation.

The child was born via non-consanguineous marriage and delivered by emergency lower (uterine) segment cesarean section (LSCS) due to meconium-stained amniotic fluid. The child cried immediately after birth. The mother's antenatal history was significant for polyhydramnios at seven months of gestation. On the third day after birth, the baby was found to have a holosystolic murmur of grade 3/6 when examined with a stethoscope. This prompted further investigation, which included an echocardiography that revealed multiple heart defects, including tetralogy of Fallot, a ventricular septal defect, a small atrial septal defect, and pulmonary atresia. Additionally, a chest X-ray did not detect the presence of a thymus, and the infant also had a submucosal cleft palate. The baby experienced a seizure episode during the second week of life and was later diagnosed with hypocalcemia, which was responsible for the seizures. Lab tests indicated low levels of parathyroid hormone and serum calcium, high levels of serum phosphorus, and normal levels of 25-hydroxyvitamin D, confirming hypoparathyroidism (Table 1).

**Table 1:** Lab parameters of the patient.

Laboratory investigation	Result	Normal level
Serum calcium	6.2	9-10.5 mg/dI
Phosphorus	7	2.4-4.1 mg/dI
Parathormone (PTH)	6	10-65 pg/mL
CD3+ (CD16+56+)	40-55%	68-82%

A medical geneticist performed further tests as part of a comprehensive medical evaluation, in which fluorescent in situ hybridization (FISH) was done to check out the TUPLE gene deletion on chromosome 22q11.2, and the diagnosis was confirmed for DGS.

Due to primary immunodeficiency and a cleft palate, the patient experienced recurrent episodes of lower respiratory tract infection (LRTI) mainly caused by *Burkholderia cepacia* and aspiration pneumonia. These conditions were managed with IV antibiotics, steroids, and frequent nebulization. However, the patient continued to suffer from recurrent infections, which necessitated long-term Ryle's tube (RT) feeds for a duration of 18 months. Despite this intervention, poor weight gain was observed in the infant, prompting a videofluoroscopic study (VFSS), which revealed a reduced suckling reflex during the oral stage. A barium swallow test did not reveal any signs of reflux or hiatus hernia. The patient was advised to have regular follow-ups every six months to monitor major infections due to a low T cell count. However, the child's poor immunity and long-term RT feeds resulted in multiple episodes of LRTI, leading to the performance of a percutaneous endoscopic gastrostomy (PEG) tube insertion at the age of 19 months. The patient was advised to continue taking prophylactic antibiotics and seek urgent care if experiencing signs of high fever or respiratory distress. After the PEG placement, the patient was gradually introduced to feeds, and once it was established that they were well-tolerated, the feeds were increased to full feeds. Subsequently, the child had fewer episodes of LRTI and aspiration pneumonia during follow-up care and gained adequate weight for his age. Definitive corrective cardiac surgery was performed when the patient achieved 10 kg of body weight at the age of 43 months. His endocrine evaluation for initial hypocalcemia and hypo-functioning parathyroid was well-maintained with adequate daily supplementation of calcium and active vitamin D.

The developmental milestones were reported to be delayed. Later in the course of his development, he showed global developmental delay with an inability to achieve head control even at six months of age, rolled over at eight months, and was only able to sit with support at the age of one year. The child learned to sit without support only at 18 months of age and started to walk alone only at 48 months. He completely lacks speech and can only make sounds and show gestures by pointing when he needs something.

At the moment, the child is receiving speech therapy, which focuses on augmentative and alternative communication strategies, including the use of sign language and picture-based communication boards, and also receiving occupational therapy to help with his gross motor skills. Despite undergoing speech and language training, there has not been any noticeable progress in his ability to speak or write words; instead, he makes sounds like hooting. On the other hand, his gross motor skills have improved with activity-based occupational therapy. The child is currently enrolled in a special school linked to the hospital's psychiatry department, but he struggles with social cues and interacting with peers.

## Discussion

The pharyngeal pouches derive from the endoderm and form within the fourth week of fetal development [7]. The third pharyngeal pouch forms the thymus and inferior region of the parathyroid gland, while the superior portion of the parathyroid gland arises from the fourth pharyngeal pouch [7]. The thymus is a lymphoid organ that aids in the development of T lymphocytes, which participate in the adaptive cellular-mediated immune response [8]. The parathyroid gland regulates calcium and phosphate concentrations via the parathyroid hormone (PTH), which acts on bones and the gastrointestinal system [9]. The classic triad of DGS includes hypocalcemia due to hypoparathyroidism, congenital cardiac anomalies, and immunodeficiency [10]. The severity of immunodeficiency ranges from mild to moderate due to either thymic hypoplasia or aplasia, respectively [11]. Hypocalcemia due to parathyroid hypoplasia manifests as paresthesia, muscle spasms, tetany, and seizures, which occurred in this patient’s second week of life [11]. This syndrome has a heterogeneous presentation, with commonly affected areas including organs of the renal, ocular, gastrointestinal, and nervous systems [10].

Approximately 90% of DGS cases result from microdeletion of the long arm (q) at locus 11.2 of chromosome 22, also known as 22q11.2 [12]. There are over 90 different genes at this locus that can be deleted, leading to DGS, including the TUP-like enhancer of split gene 1 (TUPLE1) and the T-box transcription factor 1 (TBX1) genes [12]. TBX1 stimulates the embryologic formation of the pharyngeal pouches; therefore, if affected, this leads to severe features of DGS and is commonly associated with severe defects in the heart, thymus, and parathyroid glands [12]. Studies also show that the TUPLE1 and TBX1 genes are expressed in multiple tissues, including the mesoderm of the developing brain, and microdeletion may lead to irregular neuromicrovascular formation resulting in developmental abnormalities in children [1].

Although developmental delay is a common feature of DGS, the absence of speech is not commonly seen. DGS can affect speech in two ways: (i) facial deformities such as cleft palate and lip may affect articulation and phonation or (ii) cerebral abnormalities may cause a delay in speech onset [13,14]. In this case, the patient’s cleft palate was small and due to the severity of his speech defect, it is inferred that an underlying neurological defect was most likely the cause of his lack of speech. Some speech manifestations associated with DGS include delayed speech emergence, dysarthria, velopharyngeal dysfunction, childhood apraxia of speech, and phonological disorders, all of which may persist into adolescence [10]. This is the first reported case of a complete absence of speech in a patient with DGS. The various neurological effects of DGS are not well known; however, studies show that changes in the anatomy of the brain lobes, basal ganglia, and cerebellum may lead to developmental delay. A study by Campbell et al. investigated the areas of the brain affected by DGS in 39 children with cognitive deficits using voxel-based morphometry (VBM) [15]. Results showed a significant reduction in the cerebellar gray matter as well as the white matter in the cerebellum, internal capsule, and frontal lobe [15]. The frontal lobe is responsible for voluntary movement, expressive language, and managing cognitive skills; therefore, if affected, this may lead to a delay in achieving gross and fine motor skill milestones as well as a delay or absence of speech onset [16].

Due to the risk of a decreased quality of life, a high index of clinical suspicion is required. A confirmatory diagnosis of this syndrome can be achieved with genetic testing via FISH [17]. FISH is the gold standard diagnostic tool for DGS and uses fluorescent DNA probes to evaluate for microdeletions in a specific chromosomal location [17]. It is useful in genetic counseling as well as prenatal and postnatal diagnosis [17]. After confirmation, extensive evaluation of the various organ systems is required. Tests include an echocardiogram to assess for conotruncal abnormalities, a chest X-ray to evaluate the thymic hypoplasia, T-lymphocyte panels, serum ionized calcium and phosphorus levels, PTH levels, and renal ultrasound [12]. The management of DGS is symptomatic, with the goal of preventing complications. Intravenous immunoglobulins, prophylactic antibiotics, and either a thymic or hematopoietic cell transplant can be used to manage immunodeficiency [12]. Surgical correction of life-threatening cyanotic heart diseases and cleft palate improves circulation, breathing, speech, and feeding [12]. Supplementation with calcium and vitamin D aids in the prevention of seizures and other hypocalcemic complications. Occupational and speech therapy is useful in children with developmental delays and proves beneficial in aiding children to meet their developmental milestones [12].

In this case, the patient had DGS, characterized by tetralogy of Fallot, cleft palate, thymic aplasia, and hypoparathyroidism, which led to a hypocalcemic-induced seizure. The diagnosis was confirmed with FISH, which showed the deletion of the TUPLE gene. Global developmental delay was noted in the patient as he aged; however, there was a severe speech deficit where the patient was nonverbal and showed minimal signs of improvement with speech therapy alone. This case of DGS with a complete absence of speech was a unique and unusual finding that shows the extensive range of symptoms caused by this genetic disease. A complete absence of speech could be one of the rarer symptoms of DGS, and further research into this association and the possible underlying causes is required.

## Conclusions

Developmental speech delay is a common manifestation of DGS that affects many children in various ways. Speech defects normally range from delayed onset to apraxia; however, the complete absence of speech in the setting of DGS is a new finding that should be noted. Microdeletions of the TUPLE1 and TBX1 genes on chromosome 22 are possible underlying genetic etiologies contributing to this severe symptomatic variant. Possible neurological findings in patients with complete absence of speech and DGS include reduced gray and white matter throughout the cerebrum. Early diagnosis with genetic testing and symptomatic treatment is crucial to improving the patient’s quality of life. Speech therapy is the initial management strategy for patients with speech delay; however, in some cases, such as this one, improvement may not be significant. More research needs to be conducted on the effectiveness of treatment and alternative therapies that can be utilized to improve speech outcomes. Further evaluation into the long-term outcomes for patients with DGS and the absence of speech is also required. Physicians should understand that since this disorder presents with a variety of symptoms, the treatment plan should be unique and tailored to the needs of the patient as they transition into adulthood; therefore, using a holistic multidisciplinary approach to address their medical, behavioral, and psychological needs can be beneficial.
